# How to Induce Sleep

**Published:** 1898-09

**Authors:** 


### How to Induce Sleep.

Sleep ensues when the brain is largely denuded of blood, when cerebral anemia is established. To partly empty the brain of its blood supply, to keep the head cool, the body sufficiently warm, and to send the blood rather to the lower extremities—this is the physical problem of the sleepless. It is interesting to note that during sleep a great number of the bodily functions continue quite normally without interfering with sleep itself, and therefore sleep is not so like death as some of the poets have imagined. Man asleep is not so profoundly different from man awake, the two chief points of difference, however, being these: a greater indrawing of oxygen and exhalation of carbonic acid, and a complete vaso-motor rest. The bedroom and the state of occupant (assuming the absence of external noise) are the chief factors in the problem.

The sleeping room should be airy and cool, never, for adult persons, reaching a higher temperature than 60°, though young children need greater warmth. The head should never be under the sheets, but exposed and cool. The feet should be kept warm by a little extra clothing at the foot. With a heavy sleeper there should be no thick curtains, but with a light sleeper curtains are essential, as sunlight plays upon the optic nerve and rouses that attention which it is the one object of the sleeper to keep in suspended animation. The bed should never be between fireplace and door, as it catches the draughts, and is more dangerous and more easy to contract a chill in bed than in the daytime, the especially chilly period being about 3 a. m. —*Public Health.*

